# Identification of *de novo EP300* and *PLAU* variants in a patient with Rubinstein–Taybi syndrome-related arterial vasculopathy and skeletal anomaly

**DOI:** 10.1038/s41598-021-95133-0

**Published:** 2021-08-05

**Authors:** Jong Eun Park, Eunmi Kim, Dong-Won Lee, Taek Kyu Park, Min Sun Kim, Shin Yi Jang, Jaemyung Ahn, Kwang Bo Park, Keon-Ha Kim, Hae-Chul Park, Chang-Seok Ki, Duk-Kyung Kim

**Affiliations:** 1 Department of Laboratory Medicine, Hanyang University Guri Hospital, Hanyang University College of Medicine, Guri, Republic of Korea; 2 Department of Biomedical Sciences, College of Medicine, Korea University, Ansan, Republic of Korea; 3 Division of Cardiology, Department of Medicine, Heart Vascular Stroke Institute, Samsung Medical Center, Sungkyunkwan University School of Medicine, Seoul, Republic of Korea; 4 Division of Cardiology, Department of Medicine, Samsung Changwon Hospital, Sungkyunkwan University School of Medicine, Changwon, Republic of Korea; 5 Department of Oral and Maxillofacial Surgery, Samsung Medical Center, Sungkyunkwan University School of Medicine, Seoul, Republic of Korea; 6 Department of Radiology, Samsung Medical Center, Sungkyunkwan University School of Medicine, Seoul, Republic of Korea; 7 GC Genome, Yongin, Republic of Korea

**Keywords:** Developmental biology, Genetics, Cardiology, Diseases, Medical research, Signs and symptoms

## Abstract

Rubinstein–Taybi syndrome (RSTS) is a human genetic disorder characterized by distinctive craniofacial features, broad thumbs and halluces, and intellectual disability. Mutations in the CREB binding protein (CREBBP) and E1A binding protein p300 (EP300) are the known causes of RSTS disease. EP300 regulates transcription via chromatin remodeling and plays an important role in cell proliferation and differentiation. Plasminogen activator, urokinase (PLAU) encodes a serine protease that converts plasminogen to plasmin and is involved in several biological processes such as the proteolysis of extracellular matrix-remodeling proteins and the promotion of vascular permeability and angiogenesis. Recently, we discovered a patient who presented with RSTS-related skeletal anomaly and peripheral arterial vasculopathy. To investigate the genetic cause of the disease, we performed trio whole genome sequencing of the genomic DNA from the proband and the proband’s parents. We identified two *de novo* variants coined c.1760T>G (p.Leu587Arg) and c.664G>A (p.Ala222Thr) in *EP300* and *PLAU*, respectively. Furthermore, functional loss of EP300a and PLAUb in zebrafish synergistically affected the intersegmental vessel formation and resulted in the vascular occlusion phenotype. Therefore, we hypothesize that the *de novo EP300* variant may have caused RSTS, while both the identified *EP300* and *PLAU* variants may have contributed to the patient’s vascular phenotype.

## Introduction

Rubinstein–Taybi syndrome (RSTS) is a multiple congenital anomaly syndrome characterized by distinctive craniofacial features, broad thumbs and halluces, growth retardation, and intellectual disability^1^. RSTS is an autosomal dominant disorder in humans, in which 50–60% and 8–10% of cases were caused by mutations in the CREB binding protein (CREBBP) and E1A binding protein p300 (EP300), respectively^2^.

EP300, which shares 63% homology with CREBBP at the amino acid level, encodes a large protein that consists of several KIX domains, a bromodomain, and HAT domains^3,4^. EP300 regulates transcription via chromatin remodeling and plays an important role in cell proliferation and differentiation^5,6^.

On the other hand, plasminogen activator, urokinase (PLAU) encodes a serine protease that converts plasminogen to plasmin and is involved in several biological processes such as the proteolysis of extracellular matrix-remodeling proteins and the promotion of vascular permeability and angiogenesis^7,8^. PLAU, which belongs to the peptidase S1 family, consists of a carboxyl-terminal serine protease domain and a modular amino-terminal fragment that contains a growth factor-like domain and a Kringle domain^9^. In humans, the gain-of-function defect in fibrinolysis due to the tandem duplication of the *PLAU* gene causes the development of the Quebec platelet disorder (QPD), an autosomal dominant disease^10^.

The human *EP300* and *PLAU* genes have two orthologs each in zebrafish, namely *ep300a* and *ep300b,* and *plaua* and *plaub*, respectively. The protein sequences of zebrafish EP300a/b and PLAUa/b are highly conserved with those of human EP300 and PLAU, respectively^11,12^.

In this study, we investigated the genomic DNA samples obtained from a patient with RSTS-related skeletal anomaly and peripheral arterial vasculopathy and from the patient’s parents using trio whole genome sequencing (trio WGS). Furthermore, we performed knockdown experiments in zebrafish using translation-blocking morpholinos (MOs) to determine the effects of the functional loss of EP300 and PLAU in intersegmental vessel (ISV) development.

## Results

### Clinical features

**Figure 1:**
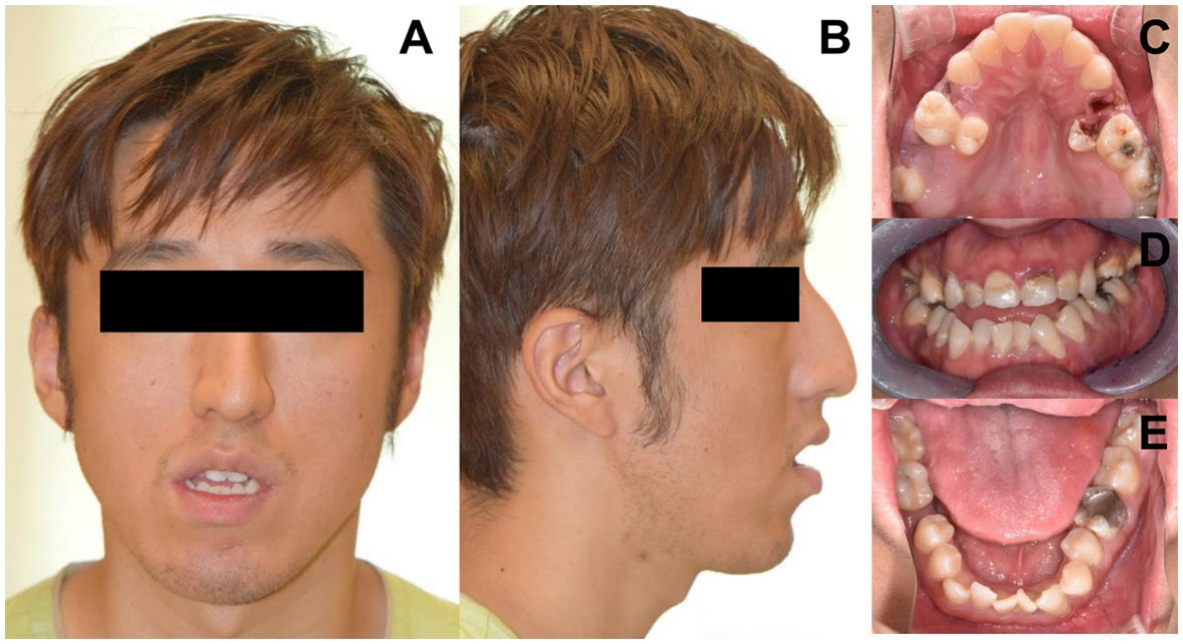
Facial and dental characteristics of the patient. The face was long and narrow and showed signs of third nerve palsy, grimacing smile, pinched and hump nose, maxillary retrognathia compared to the mandible, and skeletal Class III malocclusion without microencephaly (**A**,**B**). Dental examination revealed V-shaped and high arched palate, crowding of the teeth, anterior open bite, many dental caries, teeth fractures and ectopic eruptions, and narrow maxillary width compared to the mandible (**C**–**E**).

**Figure 2:**
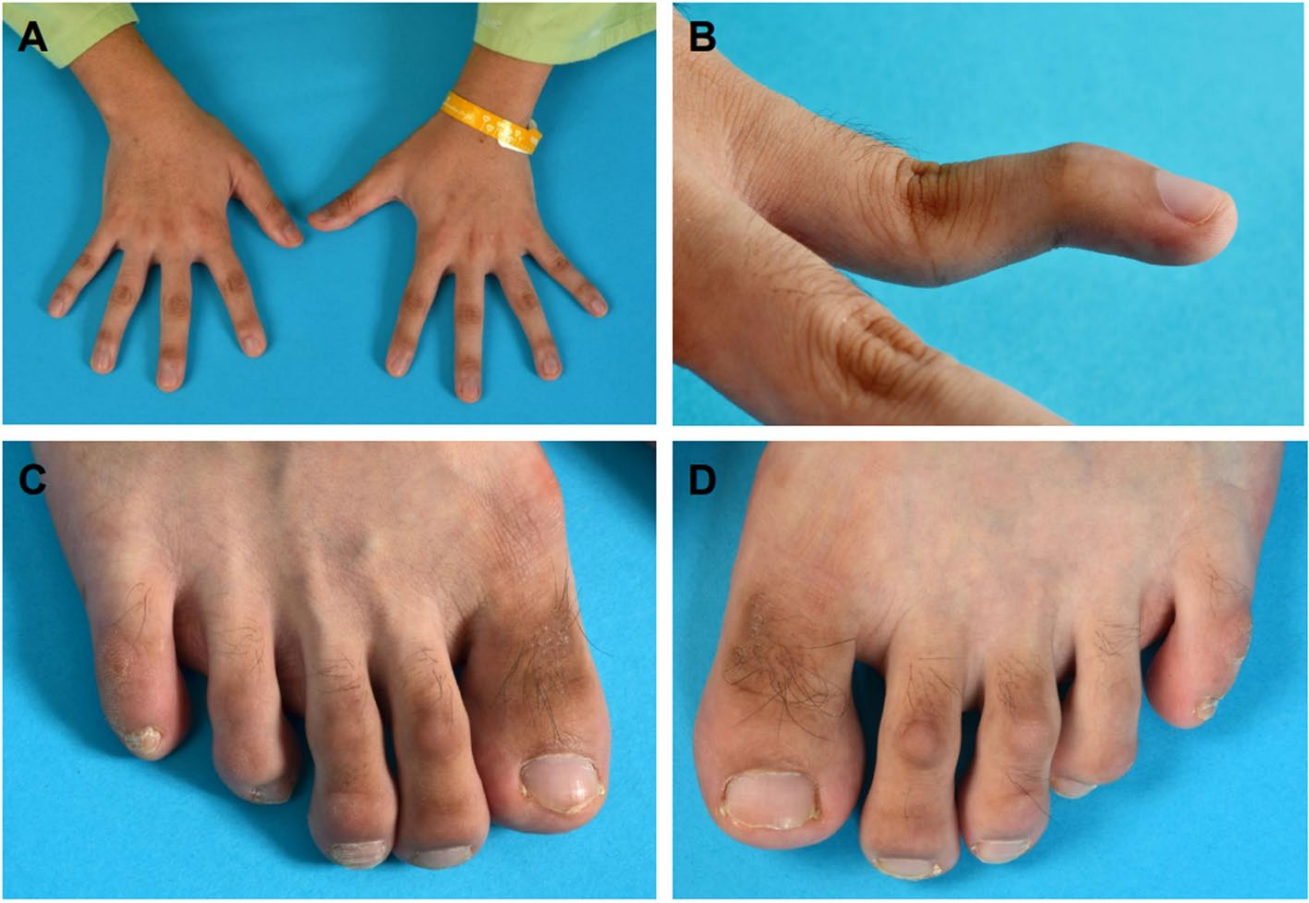
Skeletal findings of the patient. The hands showed brachydactyly (**A**) and angulation of the 5th fingers (**B**). Broad halluces and hypertrichosis were observed in the toes (**C**,**D**).

A 23-year-old man was transferred and admitted due to small amount of subarachnoid hemorrhage and right midbrain infarct. Outside hospital performed coil embolization of the right anterior inferior cerebellar artery and the perforator of right posterior cerebral artery. The patient’s face was long and narrow without microcephaly and showed signs of third nerve palsy, grimacing smile, pinched and hump nose, maxillary retrognathia compared to the mandible, and skeletal Class III malocclusion (Fig. 1). Physical examination of the extremities disclosed the brachydactyly, angulation of the 5th fingers, and broad halluces and hypertrichosis of the toes (Fig. 2). Dental examination revealed a V-shaped and high arched palate, crowding of the teeth, anterior open bite, many dental caries, teeth fractures and ectopic eruptions, and narrow maxillary width compared to the mandible (Fig. 1). Physical examination of the chest and abdomen showed no abnormalities. Past medical history disclosed he was born healthy by normal full-term delivery without preeclampsia. His postnatal growth was slow but catched up during childhood. His intellect was not impaired and he is a university student majoring in computer science. He finished mandatary military service for two years and he was in healthy condition before his illness.

**Figure 3:**
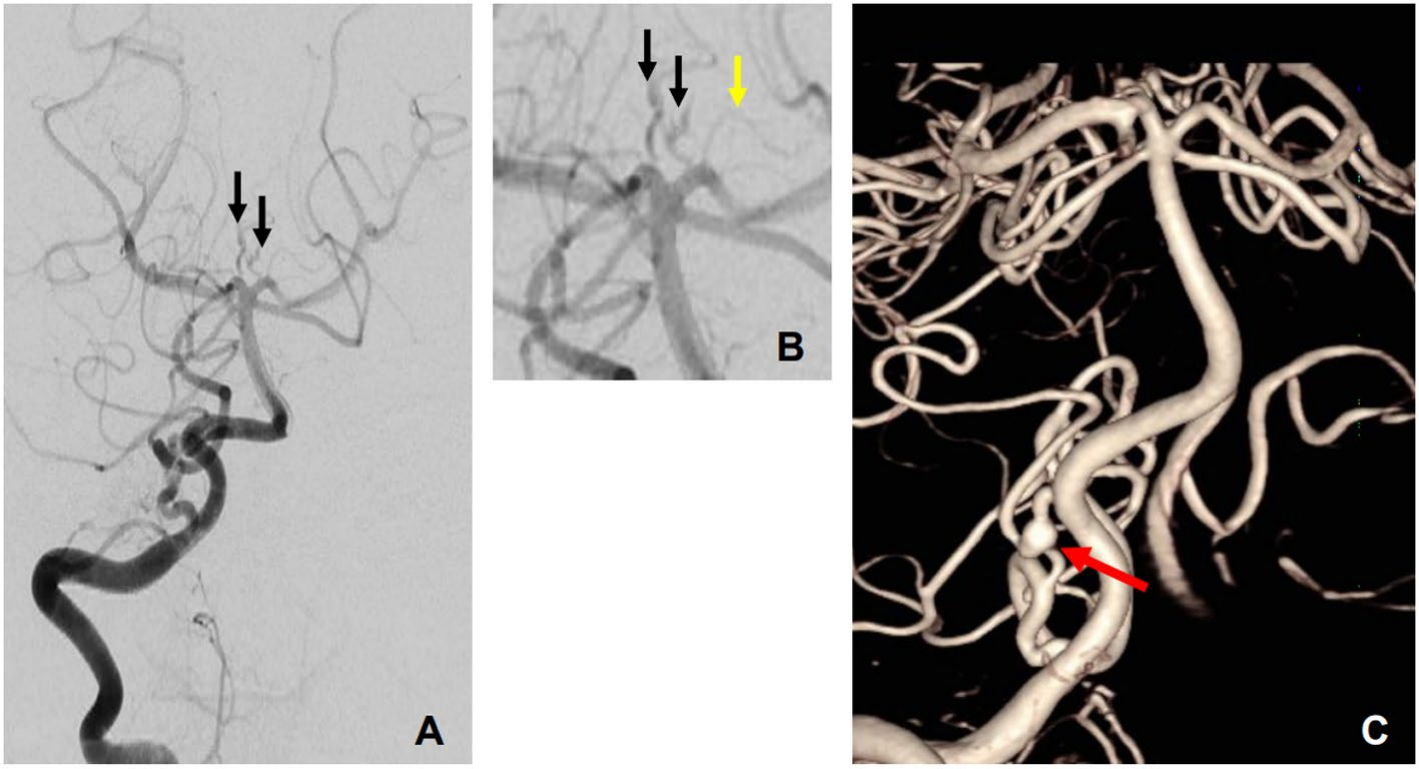
Right vertebral artery angiography of the patient. Anterior–posterior view of the right vertebral artery (**A**,**B**). Compared with the normal perforator (yellow arrow), the perforators of both posterior cerebral arteries (black arrows) were tortuous and dilated. Short segmental dilatation and aneurysm formation of the right anterior inferior cerebellar artery (red arrow) was noted.

**Figure 4:**
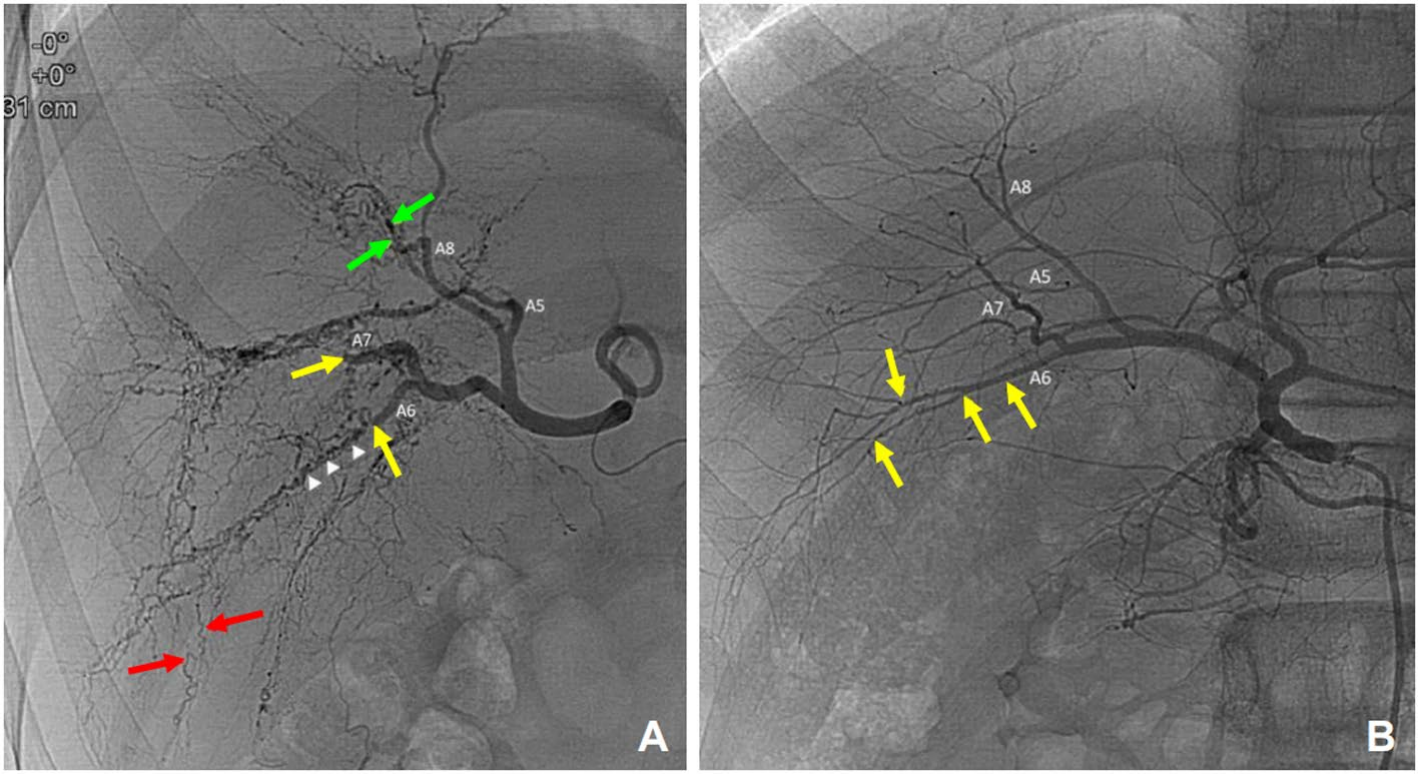
Comparison of visceral angiograms between the patient’s hepatic artery (**A**) and a normal hepatic artery (**B**). Overall, the “pruned branches” is a sign of normal peripheral hepatic arteries with extensive intrahepatic corkscrew and tortuous collateral arteries (**A**). Abrupt cut-off of second order branch of the hepatic artery types A6 and A7 (yellow arrows). Fine corkscrew collateral arteries along the A6 running course that replaces the normal A6 branch (white arrow heads). Intrahepatic tortuous collateral arteries developed in the peripheral liver (red arrows). Multiple stenosis and focal ectasia of the segmental hepatic artery (A8). This lead to the complete obstruction of the arteries and subsequent collateral development (green arrows). The normal A6 hepatic artery (**B**) showed typical smooth peripheral tapering out of the segmental hepatic artery (A6) with side-by-side ramification of the intrahepatic branches (yellow arrows).

Angiograms of the both internal carotid and right vertebral arteries from the previous outside cerebral angiography were reviewed, which revealed no vascular abnormality in the internal carotid, middle cerebral, and anterior cerebral arteries. Right vertebral angiography showed tortuous dilated perforator proximal to both posterior cerebral arteries. Furthermore, 3-dimensional angiography revealed short segmental fusiform dilatation of the right anterior inferior cerebellar artery, suggesting dissection (Fig. 3), which caused the hemorrhage. Because the brain vessels showed unusual vascular findings, we decided to perform coronary and visceral angiography. The coronary arteries had small caliber arteries with tiny aneurysms of all three branches. The splanchnic visceral angiogram revealed the “pruned branches” sign of normal peripheral hepatic arteries with extensive intrahepatic corkscrew and tortuous collateral arteries (Fig. 4). Echocardiography showed a dilated left ventricular cavity (end diastolic dimension = 58 mm, end-systolic dimension = 44 mm) with lower ejection fraction (45%). Regional wall motion abnormalities were not observed. The blood tests for liver function were normal, and no further vascular interventions were performed. The patient was discharged and prescribed with cilostazol and atenolol. No other family member had a history of similar physical findings or cardiovascular disease.

### Identification of two *de novo* missense variants in *EP300* and *PLAU*

**Figure 5:**
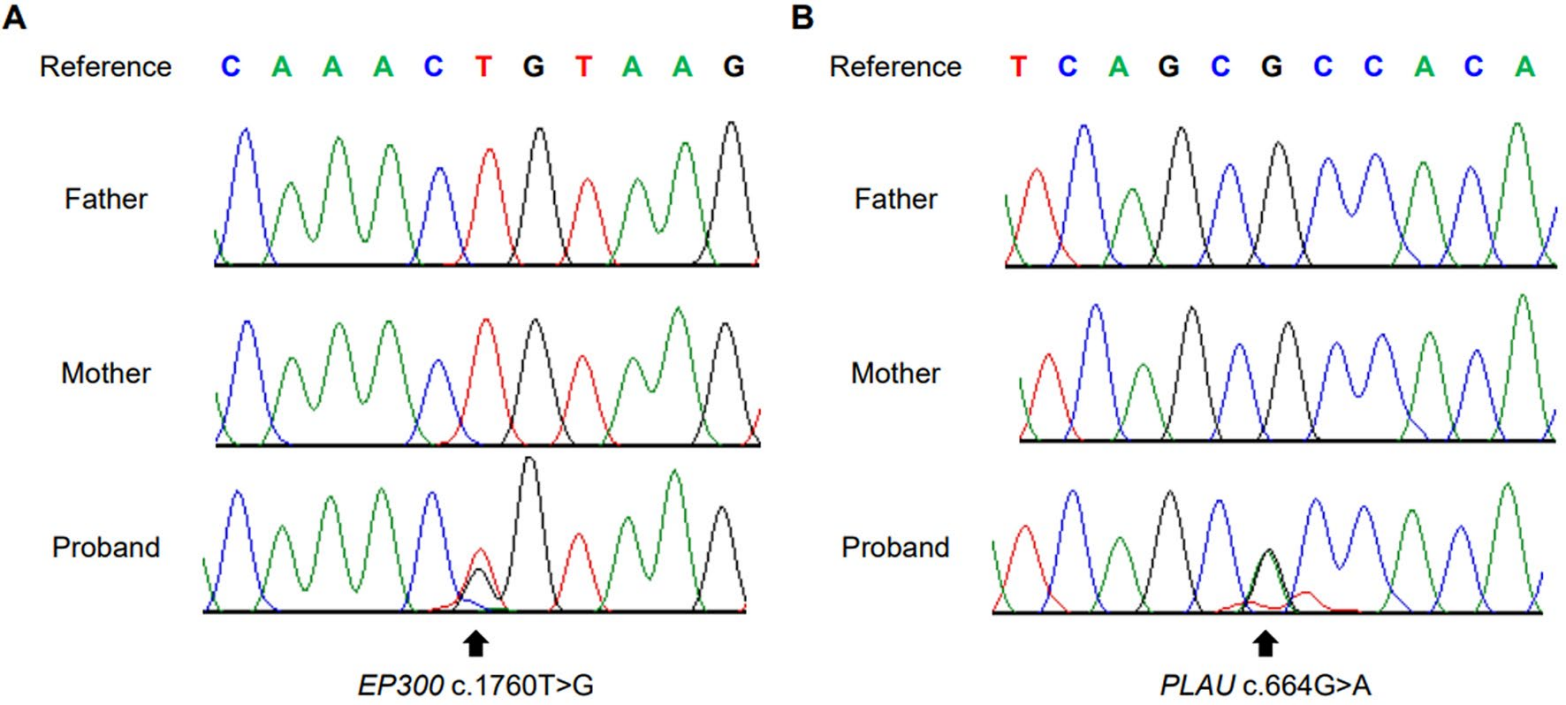
Electropherograms of *EP300* and *PLAU* obtained using Sanger sequencing. Two heterozygous missense variants in *EP300* (**A**) and *PLAU* (**B**) were identified as *de novo*.

The trio WGS analysis revealed two heterozygous *de novo* missense variants, NM_001429.3:c.1760T>G [p.Leu587Arg] and NM_002658.4:c.664G>A [p.Ala222Thr], in *EP300* and *PLAU* (Fig. 5). Subsequently, Sanger sequencing was performed to validate these variants. According to the 2015 American College of Medical Genetics and Genomics and the Association for Molecular Pathology guideline (2015 ACMG AMP guideline)^13^, *EP300* c.1760T>G variants was classified as likely pathogenic variant (LPV). The *EP300* c.1760T>G variant, which is located in the last base before the splice site of the KIX domain, was not registered in any public database. According to in silico analysis, this variant was predicted to have no effect on splicing in MaxEntScan and SpliceAI, but was predicted to be deleterious in SIFT, Polyphen2, MutationTaster, PROVEAN, and REVEL. The *PLAU* c.664G>A variant was classified as LPV, which was located in the peptidase S1 domain was found in gnomAD and had a minor allele frequency of 0.0000758. The *PLAU* c.664G>A variant was predicted to be deleterious in in silico analysis using SIFT, Polyphen2, MutationTaster, PROVEAN, and REVEL.

### Loss-of-function of *ep300a* and *plaub* causes vascular occlusion in the ISVs of zebrafish

**Figure 6:**
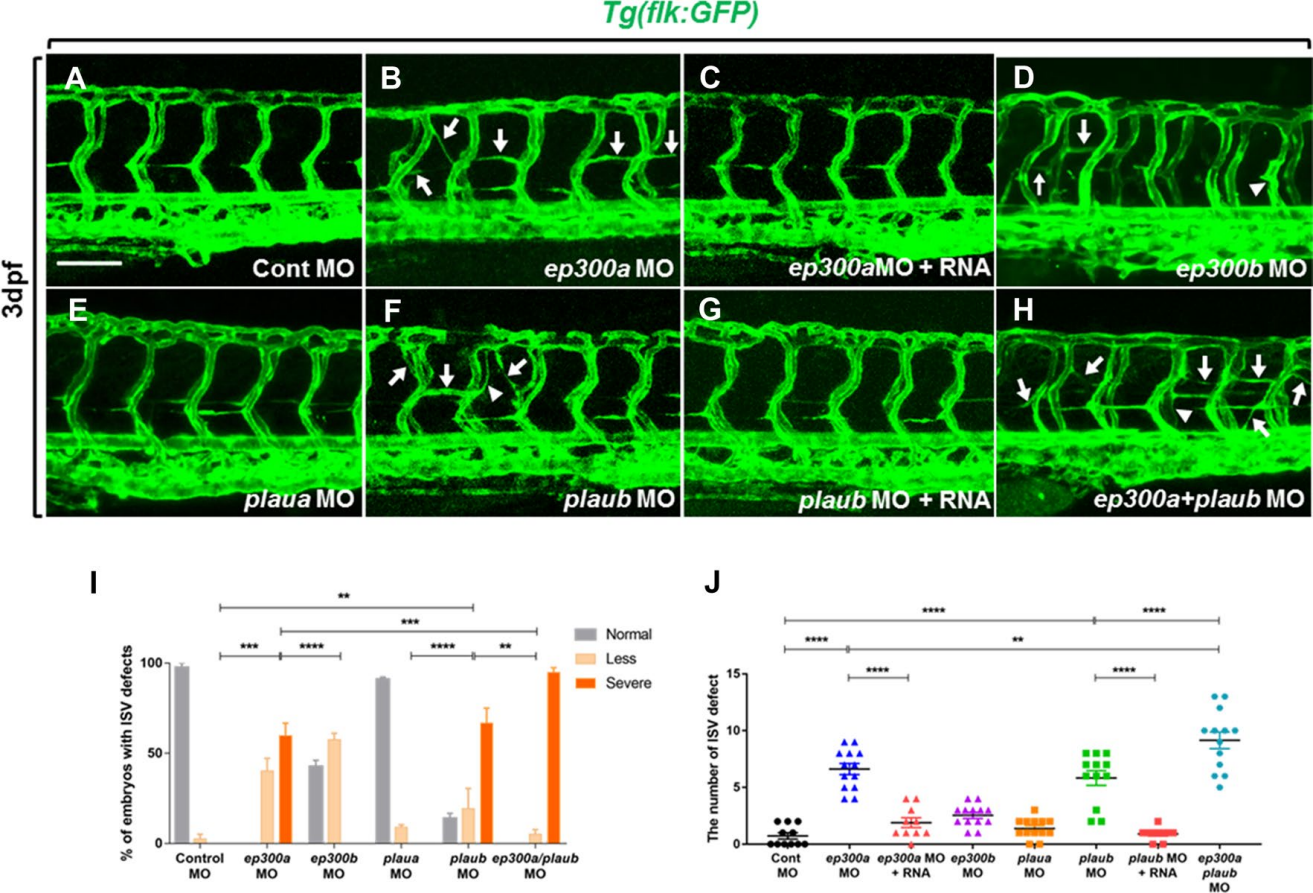
Knockdown of *ep300a* and *plaub* caused impaired intersegmental vessel (ISV) formation in zebrafish. Lateral views of the trunk of *Tg*(*flk:gfp*) zebrafish larvae (dorsal or ‘to the top’ and anterior or ‘to the left’) at 3 days post-fertilization (dpf) (**A**–**H**). Arrows indicate ISVs with abnormal branch formation. Arrowheads indicate ISVs with abnormal shape. Quantification of the number of embryos with ISV defects (**I**) in the control, *ep300a*, *ep300b*, *plaua*, and *plaub* morpholino (MO)-injected larvae at 3 dpf ([Control MO], normal: 97.43%, less: 2.57%, severe: 0% (n = 34); [*ep300a* MO], normal: 0% less: 40.27%, severe: 59.73%, (n = 37); [*ep300b* MO], normal: 43.25%, less: 56.75%, severe: 0%, (n = 37); [*plaua* MO], normal: 90.97%, less: 9.03%, severe: 0%, (n = 36); [*plaub* MO], normal: 11.43%, less: 8.57%, severe: 80%, (n = 35); [*ep300a*/*plaub* MO], normal: 0%, less: 5.41%, severe: 94.59%, (n = 37)). Quantification of defective ISVs (**J**) in the control, *ep300a*, *ep300b*, *plaua*, and *plaub* MO-injected larvae and *ep300a* MO + RNA and *plaub* MO + RNA-injected larvae at 3 dpf (Control MO: 0.72 ± 0.27 (n = 11), *ep300a* MO: 6.62 ± 0.49 (n = 13), *ep300a* MO + RNA: 1.9 ± 0.43 (n = 10), *ep300b* MO: 2.54 ± 0.27 (n = 13), *plaua* MO: 1.39 ± 0.24 (n = 13), *plaub* MO: 5.83 ± 0.65 (n = 12), *plaub* MO + RNA: 0.9 ± 0.18 (n = 10), *ep300a*/*plaub* MO: 9.15 ± 0.73 (n = 13). **p < 0.01, ***p < 0.01, ****p < 0.0001; scale bar = 100 µm).

To investigate the loss-of-function of *ep300a/b* and *plaua/b* in zebrafish, we performed knockdown experiments using translation-blocking MOs to target *ep300a* (*ep300a* MO), *ep300b* (*ep300b* MO), *plaua* (*plaua* MO), and *plaub* (*plaub* MO). To test the specificity of each MO, we first injected each MO (together with heat shock-inducible plasmid constructs, which expressed mCherry-tagged EP300a/b or PLAUa/b proteins) into wild-type embryos. We confirmed that mCherry expression was inhibited in *ep300a* MO, *ep300b* MO, *plaua* MO, and *plaub* MO-injected embryos (Supplementary Fig. 1). We then injected the MOs into *Tg*(*flk:gfp*) larvae that express GFP fluorescent proteins in the blood vessels, including ISVs^14^. Results showed abnormal ISV phenotype with ectopic branch and abnormal vessel formation in each morphant (Fig. 6). There were more severe ISV defects observed in *ep300a* (severe, 22/37; less, 15/37; and normal, 0/37; where n = 37) and *plaub* morphants (severe, 28/35; less, 3/35; and normal, 4/35; where n = 35) than in *ep300b* (severe, 0/37; less, 21/37; normal, 16/37; where n = 37) and *plaua* morphants (severe, 0/36; less, 4/36; and normal, 32/36; where n = 36) (Fig. 6A,B,D–F,I,J), and ISV defects caused by the injection of *ep300a* MO and *plaub* MO were rescued by co-injection with *ep300a* and *plaub* mRNAs, respectively (Fig. 6C,G,J). These data indicate that EP300a and PLAUb are the major isoforms required for normal blood vessel development. Notably, injection of both *ep300a* and *plaub* MOs into *Tg*(*flk:gfp*) embryos resulted in more severe ISV defects compared to either *ep300a* or *plaub* morphants (*ep300a/plaub* MO: severe, 35/37; less, 2/37; and normal, 0/37; where n = 37) (Fig. 6H–J), indicating that both EP300a and PLAUb are required for normal ISV development and that the functional loss of both proteins synergistically causes defects during blood vessel development.

**Figure 7:**
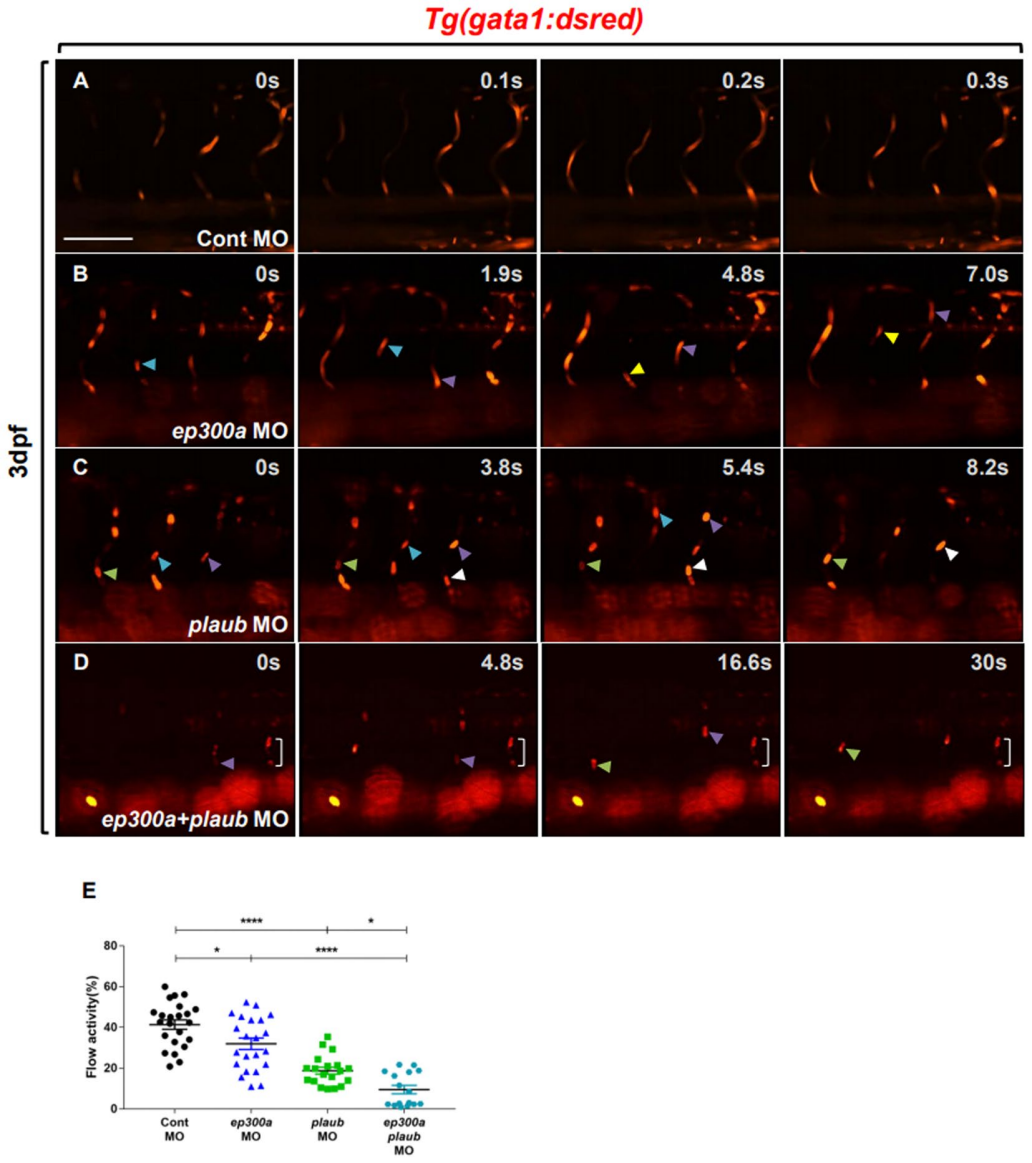
Knockdown of *ep300a* and *plaub* caused the vascular occlusion phenotype in zebrafish. Lateral views of the trunk of *Tg*(*gata1:dsred*) zebrafish larvae (dorsal or ‘to the top’ and anterior or ‘to the left’) at 3 days post-fertilization (dpf) (**A**–**D**). Arrowheads indicate the slowly circulating red blood cells (RBCs) in the intersegmental vessels (ISVs). The bracketed area indicates stalled RBCs in the ISVs. Analysis of RBC circulation (**E**) in the control, *ep300a*, and *plaub* morpholino (MO)-injected larvae at 3 dpf (Control MO: 41.39 ± 2.39 (n = 23), *ep300a* MO: 31.97 ± 2.78 (n = 22), *plaub* MO: 18.79 ± 1.68 (n = 19), *ep300a*/*plaub* MO: 9.50 ± 2.01 (n = 16); *p < 0.05, ****p < 0.0001; scale bar = 100 µm).

To verify whether the functional loss of EP300a or PLAUb caused a synergistic effect on vascular occlusion, we injected *ep300a*, *plaub*, or *ep300a* + *plaub* MOs into *Tg*(*gata1:dsred*) embryos that express RFP fluorescent protein in the red blood cells (RBCs)^15^, and recorded the blood flow activity in ISVs for 30 s at 3 days post-fertilization (dpf). We observed that the blood flow activity was significantly reduced in *ep300a* or *plaub* morphants compared to the control morphants (Fig. 7A–C,E; Supplementary Videos 2–4). Notably, we confirmed that the blood flow activity in ISVs was significantly decreased in *ep300a*/*plaub* morphants compared to *ep300a* or *plaub* morphants (Fig. 7B–E; Supplementary Videos 3–5). These results demonstrate that both EP300a and PLAUb are required for normal ISV formation. Thus, the functional loss of these proteins synergistically affected ISV formation and resulted in the vascular occlusion phenotype in zebrafish.

## Discussion

RSTS is mainly caused by the mutations in two paralogs, CREBBP and EP300, which commonly act as transcriptional co-activators of other transcription factors^16^. Although the disease caused by these gene variants has similar clinical symptoms, patients with *EP300*-related RSTS—86% of whom possess loss-of-function variants, truncating variants, and small rearrangements—exhibit fewer facial deformities and mild intellectual disability compared to those with CREBBP-related RSTS^17^. In this study, the *EP300* c.1760T>G variant was located at the last base before the canonical splicing site, but in silico analysis predicting splicing was predicted to have a low probability of affecting splicing. However, c.1760T>G variant was also predicted deleterious in missense predictions in in silico analysis, the possibility of affecting protein function as a missense variant may also be considered. The skeletal abnormalities observed in our study patient were milder than those observed in the typical RSTS symptoms, which we infer to be associated with the identified *EP300* variant. Also, neither mental retardation nor growth retardation was noted.

Vascular-related symptoms are quite rare in RSTS patients. However, cerebrovascular abnormalities, such as spontaneous dissection of supraaortic arteries and dissecting aneurysms of the anterior cerebral artery, have been reported in some patients^18,19^. Mice with a homozygous mutation in CREBBP were reported to exhibit impaired vasculo-angiogenesis^20^. However, no vascular defects such as those observed in the present study have been reported in *EP300*-related RSTS patients or other vertebrates.

QPD, a well-known disease caused by the mutation in *PLAU*, is characterized by the platelet-dependent gain-of-function defect in fibrinolysis^21^. In addition, *PLAU* is related to the increased risk of developing Alzheimer's disease^22^ and is known to be involved in cancer cell migration^23^. Several studies have reported that the urokinase system composed of PLAU and its receptor is associated with the differentiation and proliferation of smooth muscle and endothelial cells during vascular remodeling^24,25^. Furthermore, the urokinase system is related to the migration of vascular cells and the formation of capillary-like branched structures^26^. A study in mice revealed that urokinase deficiency promoted aneurysm rupture, which may be associated with the inability to resolve the transmural thrombi within the aneurysmal tissue^27^. In this study, the patient had artery aneurysm, occlusions, and plexiform collaterals in the small- to medium-sized arteries. Although it was not confirmed if the *PLAU* c.664G>A identified in our subject was a loss-of-function variant, the results of our zebrafish experiments and those of the previous mouse study suggest the possibility of PLAU-associated vasculopathy in humans.

In conclusion, two *de novo* variants were discovered in the *EP300* and *PLAU* genes of a patient with RSTS-related skeletal anomaly and characteristic peripheral arterial vasculopathy. Genetic investigation using the zebrafish model revealed that the functional loss of EP300 and PLAU can cause abnormalities during blood vessel formation and synergistically affect vascular occlusion. However, further research is needed to confirm the synergistic relationship between EP300 and PLAU and elucidate the mechanisms involved. Finally, we hypothesize that the *de novo EP300* variant may have caused RSTS, while both the *EP300* and *PLAU* variants may have contributed to the vascular phenotype of our study patient.

## Methods

### Study participants

The subjects were evaluated by history taking, clinical examination, echocardiography, magnetic resonance imaging (MRI) and MR angiography of the brain, and angiography of the brain and visceral organs.

This study was performed in accordance with the Declaration of Helsinki and was approved by the Institutional Review Board of Samsung Medical Center (SMC 2016-11-039). Informed consent from the patient and patient's parents were obtained for clinical and genetic investigations, and publishing patient pictures and informations.

### Trio WGS and data analysis

Genomic DNA samples isolated from the peripheral blood of the proband and the proband’s parents were used for trio WGS. To narrow down the number of candidate variants, variants with minor allele frequency > 0.01 were removed from the Genome Aggregation Database (gnomAD; http://gnomad.broadinstitute.org/) and then filtered using a minimum read depth of coverage < 10 ×. Filtered variants were interpreted using the 2015 ACMG AMP guideline^13^.

### Zebrafish lines and morpholino microinjection

Wild-type AB, *Tg*(*flk:gfp*)^14^ and *Tg*(*gata1:dsred*)^15^ zebrafish of either sex were used in this study. The translation-blocking MOs, which target the start codons of *ep300a*, *ep300b*, *plaua*, and *plaub* mRNAs, and the standard control MO were purchased from Gene-Tools, LLC (Philomath, OR, USA). The MO specificity was verified using heat shock-inducible gene expression analysis. The MO sequences were as follows: *ep300a* MO (5′-ACGTTCTCGGCCATATTTTTTAACG-3′), *ep300b* MO (5′-GGAGTCCAGCACATTATCGGCCATA-3′), *plaua* MO (5′-ACCCCCTAGTATACACTTCATCTTG-3′), *plaub* MO (5′-GAATTCCAGACATCTCGCCTCTACC-3′), and standard control MO (5′-CCTCTTACCTCAGTTACAATTTATA-3′). Varied concentrations of antisense oligonucleotides (MO) were injected into the yolk of each embryo at one-cell stage: 7.6 ng for the *ep300a* MO, 10 ng for the *ep300b* MO, 10 ng for the *plaua* MO, 8.5 ng for the *plaub* MO, and 8 ng for the *ep300a*/*plaub* MO in phenol red and 0.1 M potassium chloride.

### Plasmid construction

To construct heat shock-inducible plasmids containing the *ep300a*, *ep300b*, *plaua*, or *plaub* ORFs, we amplified the respective gene ORF without a stop codon using reverse-transcription polymerase chain reaction (RT-PCR). The amplified PCR products were cloned into a middle entry vector using BP Clonase II (Invitrogen, Carlsbad, CA, USA). Then, the middle entry vectors containing *ep300a*, *ep300b*, *plaua*, or *plaub* ORFs were recombined with a 5′ entry clone containing a fragment of heat shock protein 70 (*Hsp70*) promoter, a 3′ entry clone containing the *mcherry*-*polyA* gene, and a pDestTol2pA2 from the tol2kit using LR Clonase II (Invitrogen). The sequences of designed primers were as follows: *ep300a* attB1 forward (5′-GGGGACAAGTTTGTACAAAAAAGCAGGCTCCCGTTAAAAAATATGGCCGAGAACGT-3′), *ep300a* attB2 reverse (5′-GGGGACCACTTTGTACAAGAAAGCTGGGTCGTGTTGAGGTGCGGGCCCTCCTGGC-3′), *ep300b* attB1 forward (5′-GGGGACAAGTTTGTACAAAAAAGCAGGCTCCTATGGCCGATAATGTGCTGGACTCC-3′), *ep300b* attB2 reverse (5′-GGGGACCACTTTGTACAAGAAAGCTGGGTCGCCCATTCTAGGTGCACCATTCATC-3′), *plaua* attB1 forward (5′-GGGGACAAGTTTGTACAAAAAAGCAGGCTCCCAAGATGAAGTGTATACTAGGGGGT-3′), *plaua* attB2 reverse (5′-GGGGACCACTTTGTACAAGAAAGCTGGGTCGACCCAGCATGGAGTGATGAGGGTT-3′), *plaub* attB1 forward (5′-GGGGACAAGTTTGTACAAAAAAGCAGGCTCCGGTAGAGGCGAGATGTCTGGAATTC-3′), and *plaub* attB2 reverse (5′-GGGGACCACTTTGTACAAGAAAGCTGGGTCTACCTTCATGCTCCGCCCCTCTGCC-3′). To produce pCS2-*ep300a* and pCS2-*plaub* constructs, KAT domain of *ep300a* and full length of *plaub* were amplified by RT-PCR using 24hpf cDNA with designed primers. The amplified PCR products were cloned into pCS2 + vector linearized by SmaI. The sequences of designed primers were as follows: *ep300a* Kozak KAT forward (5′-GCCACCATGGGCAAAGAGAATAAATATGCTGC-3′), *ep300a* KAT reverse (5′-AGGCCTTCAGCACTCATTACAGGTATAG-3′), *plaub* forward (5′-ATGTCTGGAATTCTCGTGTGG-3′) and *plaub* reverse (5′-TCATGAGAGTGAGGTCAGGC-3′).

### Heat-shock induction

To verify the specificity of MO using heat shock-inducible gene expression, the *ep300a*, *ep300b*, *plaua*, or *plaub* MOs (together with each corresponding heat-shock inducible plasmid) were injected into each of the embryos of wild-type AB at one-cell stage. Injected embryos were heat-shocked at 20 h post-fertilization (hpf) to induce mCherry expression and were then analyzed at 30 hpf.

### The mRNA synthesis and microinjection of *ep300a* and *plaub*

For in vitro transcription of *ep300a* and *plaub* mRNA, pCS2-*ep300a* and pCS2*-plaub* constructs were linearized using NotI restriction enzymes and transcribed using the mMESSAGE mMACHINE SP6 Transcription kit (Invitrogen). The one-cell stage *Tg*(*flk:gfp*) embryos were co-injected with 15 ng of synthesized *ep300a* mRNA and 7.6 ng of *ep300a* MO, or 15 ng of *plaub* mRNA and 8.5 ng of *plaub* MO in phenol red and 0.1 M potassium chloride.

### In vivo fluorescence imaging and statistical analysis

For in vivo imaging of ISVs in live zebrafish, the embryos were anesthetized with Tricaine (Sigma-Aldrich, St. Louis, MO, USA), embedded in 1.5% low-melting agarose, and viewed under Nikon A1Si confocal microscope (Nikon Instruments Inc., Tokyo, Japan). Blood flow activity was recorded for 30 s, and the recorded files were analyzed using DanioScope (Noldus Information Technology, Wageningen, The Netherlands). All statistical data were analyzed using GraphPad Prism 7 software (GraphPad, San Diego, CA, USA). One-way ANOVA, followed by Tukey’s multiple comparison test, was used for analyzing the number of defective ISVs and blood flow activity. Statistic Graphs are expressed as the mean ± standard error of the mean (SEM). The level of significance was set to *p*-value < 0.05.

### Ethics approval

All experimental procedures were approved by the Korea University Institutional Animal Care and Use Committee (IACUC) and conducted in accordance with the animal experimental guidelines of the Korea National Veterinary Research and Quarantine Service. The study was conducted in compliance with the ARRIVE guidelines.

## Supplementary Information

Supplementary Figure 1.

Supplementary Video 2.

Supplementary Video 3.

Supplementary Video 4.

Supplementary Video 5.

Supplementary Captions.

## Data Availability

The data that support the findings of this study are available on request from the corresponding author.

## References

[1] Hennekam RC (2006). Rubinstein–Taybi syndrome. Eur. J. Hum. Genet..

[2] Stevens, C. A. Rubinstein–Taybi syndrome. In *GeneReviews®* (eds Adam, M. P. *et al.*) (University of Washington, 1993–2021). 20301699

[3] Giles RH, Peters DJ, Breuning MH (1998). Conjunction dysfunction: CBP/p300 in human disease. Trends Genet..

[4] Delvecchio M, Gaucher J, Aguilar-Gurrieri C, Ortega E, Panne D (2013). Structure of the p300 catalytic core and implications for chromatin targeting and HAT regulation. Nat. Struct. Mol. Biol..

[5] Gayther SA (2000). Mutations truncating the EP300 acetylase in human cancers. Nat. Genet..

[6] Goodman RH, Smolik S (2000). CBP/p300 in cell growth, transformation, and development. Genes Dev..

[7] Danø K (2005). Plasminogen activation and cancer. Thromb. Haemost..

[8] Nassar T (2011). Urokinase plasminogen activator regulates pulmonary arterial contractility and vascular permeability in mice. Am. J. Respir. Cell Mol. Biol..

[9] Huai Q (2006). Structure of human urokinase plasminogen activator in complex with its receptor. Science.

[10] Blavignac J, Bunimov N, Rivard GE, Hayward CP (2011). Quebec platelet disorder: Update on pathogenesis, diagnosis, and treatment. Semin. Thromb. Hemost..

[11] Babu A (2018). Chemical and genetic rescue of an ep300 knockdown model for Rubinstein Taybi Syndrome in zebrafish. Biochim. Biophys. Acta Mol. Basis Dis..

[12] Bager R (2012). Urokinase-type plasminogen activator-like proteases in teleosts lack genuine receptor-binding epidermal growth factor-like domains. J. Biol. Chem..

[13] Richards S (2015). Standards and guidelines for the interpretation of sequence variants: A joint consensus recommendation of the American College of Medical Genetics and Genomics and the Association for Molecular Pathology. Genet. Med..

[14] Jin SW, Beis D, Mitchell T, Chen JN, Stainier DY (2005). Cellular and molecular analyses of vascular tube and lumen formation in zebrafish. Development.

[15] Traver D (2003). Transplantation and in vivo imaging of multilineage engraftment in zebrafish bloodless mutants. Nat. Immunol..

[16] Ogryzko VV, Schiltz RL, Russanova V, Howard BH, Nakatani Y (1996). The transcriptional coactivators p300 and CBP are histone acetyltransferases. Cell.

[17] Fergelot P (2016). Phenotype and genotype in 52 patients with Rubinstein–Taybi syndrome caused by EP300 mutations. Am. J. Med. Genet. A.

[18] Fischer S, Bäzner H, Henkes H (2013). Cervical artery dissection in a young patient with Rubinstein–Taybi syndrome. Clin. Neuroradiol..

[19] Ishizaka S (2010). Dissecting aneurysm of the anterior cerebral artery with Rubinstein-Taybi syndrome—A case report. Brain Nerve.

[20] Oike Y (1999). Mice homozygous for a truncated form of CREB-binding protein exhibit defects in hematopoiesis and vasculo-angiogenesis. Blood.

[21] Paterson AD (2010). Persons with Quebec platelet disorder have a tandem duplication of PLAU, the urokinase plasminogen activator gene. Blood.

[22] Finckh U (2003). Association of late-onset Alzheimer disease with a genotype of PLAU, the gene encoding urokinase-type plasminogen activator on chromosome 10q22.2. Neurogenetics.

[23] Duffy MJ (2004). The urokinase plasminogen activator system: Role in malignancy. Curr. Pharm. Des..

[24] Parfyonova YV, Plekhanova OS, Tkachuk VA (2002). Plasminogen activators in vascular remodeling and angiogenesis. Biochem. Biokhimiia.

[25] Menshikov M (2006). Urokinase plasminogen activator stimulates vascular smooth muscle cell proliferation via redox-dependent pathways. Arterioscler. Thromb. Vasc. Biol..

[26] Semina E (2016). The role of urokinase in vascular cell migration and in regulation of growth and branching of capillaries. Cell Tissue Biol..

[27] Uchida HA, Poduri A, Subramanian V, Cassis LA, Daugherty A (2011). Urokinase-type plasminogen activator deficiency in bone marrow-derived cells augments rupture of angiotensin II-induced abdominal aortic aneurysms. Arterioscler. Thromb. Vasc. Biol..

