# Supplementary figures and images for: Identification of de novo EP300 and PLAU variants in a patient with Rubinstein–Taybi syndrome-related arterial vasculopathy and skeletal anomaly
Source: Sci Rep. 2021 Aug 5;11:15931. doi: 10.1038/s41598-021-95133-0 (PMC8342626; doi:10.1038/s41598-021-95133-0)

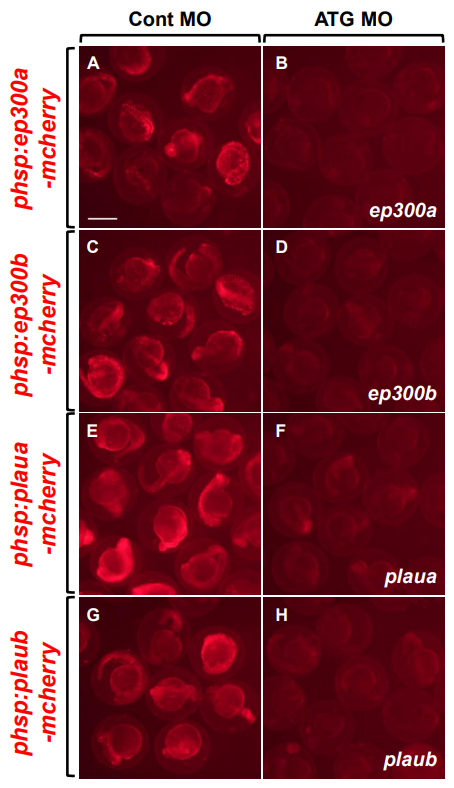

Supplement: Supplementary file 1 — Supplementary Figure 1. [file 41598_2021_95133_MOESM1_ESM.tif]
