# Supplementary material for: Identification of de novo EP300 and PLAU variants in a patient with Rubinstein–Taybi syndrome-related arterial vasculopathy and skeletal anomaly
Source: Sci Rep. 2021 Aug 5;11:15931. doi: 10.1038/s41598-021-95133-0 (PMC8342626; doi:10.1038/s41598-021-95133-0)
Supplement: Supplementary file 6 — Supplementary Captions. [file 41598_2021_95133_MOESM6_ESM.docx]

**Supplementary information**

**Supplementary Figure 1. Efficiency test of translation-blocking morpholinos (MOs) against *ep300a*, *ep300b*, *plaua*, and *plaub*.** Whole-mount images of MO-injected zebrafish embryos with heat shock-inducible plasmids at 30 h post-fertilization (hpf) (**A–H**). Embryos were heat-shocked at 20 hpf to induce *mcherry* expression. The heat shock-induced *mcherry* expression was inhibited in *ep300a*, *ep300b*, *plaua*, and *plaub* MO-injected embryos (scale bar = 2,500 µm)

**Supplementary Video 2. Blood flow in the intersegmental vessels (ISVs) of control morpholino (MO)-injected zebrafish larvae.** Lateral view of the trunk of *Tg(gata1:dsred)* zebrafish larvae (dorsal to the top and anterior to the left) at 3 days post-fertilization (dpf). The movie shows the normal blood circulation in the ISVs of control MO-injected larvae

**Supplementary Video 3. Blood flow in the intersegmental vessels (ISVs) of *ep300a* morpholino (MO)-injected zebrafish larvae.** Lateral view of the trunk of *Tg(gata1:dsred)* zebrafish larvae (dorsal to the top and anterior to the left) at 3 days post-fertilization (dpf). The movie shows the slow blood circulation in the ISVs of *ep300a* MO-injected larvae

**Supplementary Video 4. Blood flow in the intersegmental vessels (ISVs) of *plaub* morpholino (MO)-injected zebrafish larvae**. Lateral view of the trunk of *Tg(gata1:dsred)* zebrafish larvae (dorsal to the top and anterior to the left) at 3 days post-fertilization (dpf). The movie shows the slow blood circulation in the ISVs of *plaub* MO-injected larvae

**Supplementary Video 5. Blood flow activity in the intersegmental vessels (ISVs) of *ep300a*/*plaub* morpholino (MO)-injected zebrafish larvae**. Lateral view of the trunk of *Tg(gata1:dsred)* zebrafish larvae (dorsal to the top and anterior to the left) at 3 days post-fertilization (dpf). The movie shows the extremely slow and stalled blood circulation in the ISVs of *ep300a*/*plaub* MO-injected larvae
